# Repeated thrombus occlusion in superficial femoral artery at the gap between two stents: a case report

**DOI:** 10.1093/ehjcr/ytad542

**Published:** 2023-11-06

**Authors:** Yuki Matsumoto, Ryutaro Shimada, Hidemi Morioka, Yoshihiro Morino

**Affiliations:** Division of Cardiology, Ofunato Hospital, Ofunato City, Iwate, Japan; Division of Cardiology, Ofunato Hospital, Ofunato City, Iwate, Japan; Division of Cardiology, Ofunato Hospital, Ofunato City, Iwate, Japan; Division of Cardiology, Iwate Medical University, Morioka, Iwate, Japan

**Keywords:** Acute limb ischaemia, Stents gap, Paclitaxel-eluting stent, Interwoven stent, Endovascular treatment

## Abstract

**Background:**

In recent years, endovascular treatment has emerged as a preferred option for treating long lesions in the superficial femoral artery (SFA), including those classified as Trans-Atlantic Inter-Society Consensus IIC and D. This approach may involve the use of multiple stents to ensure adequate coverage of the entire lesion, as maintaining primary patency is a key consideration in the treatment strategy.

**Case summary:**

An 82-year-old woman underwent endovascular treatment with two stents for a chronic total occlusion lesion in the left SFA. Six months later, she was admitted to our hospital with acute limb ischaemia (ALI). Angiography revealed significant thrombus within the stents and a gap between the stents, while intravascular ultrasounds showed neointimal hyperplasia at the gap. Initially, the patient was treated with a cutting balloon for the gap, but experienced another episode of ALI the following day. Subsequently, a stent was placed to cover the gap, resulting in the resolution of ALI without further recurrence.

**Discussion:**

Superficial femoral arteries expose the stent to high stresses due to the unique external forces. When multiple stents are implanted, there must be sufficient overlap. If a stent gap occurs, stent deployment is unavoidable due to the neointimal hyperplasia as well as the coronary stent gap. Further research and clinical expertise are needed to optimize stent placement strategies and minimize stent-related complications in SFA lesions.

**Learning points**

- Stent gaps should be avoided or minimized to prevent neointimal hyperplasia and acute limb ischaemia (ALI), by ensuring sufficient overlap during placement
- Stenting may be considered as a potential intervention for neointimal hyperplasia to prevent ALI.

## Introduction

Endovascular treatment is becoming a common therapy for lower extremity artery disease (LEAD). Endovascular treatment is now recommended for lesions <25 cm in either occlusion or stenosis, unless there is a history of previous endovascular treatment.^1^ Long-term results are considered to be good when the stent is implanted in such a way that the lesion is adequately pre-dilated with a balloon to ensure complete coverage of the stent.^2^ Existing peripheral vascular stents are limited to a maximum length of 15 cm, and in many cases, two or more stents are overlapped and implanted.

## Summary figure

**Table:** 

Time	Events
6 months ago	Overlapped paclitaxel-eluting stent (PES) and interwoven stent (IWS) were implanted in the left popliteal artery to the middle superficial femoral artery (SFA).
Initial presentation	The patient complained the resting leg pain for 2 days and revealed total occlusion of her SFA by a computed tomography scan. The diagnosis was acute limb ischaemia (ALI) and angiography showed total occlusion of the ostial SFA and separation of the PES from the IWS. Endovascular therapy (EVT) was successfully performed with thrombus aspiration and balloon angioplasty.
The next morning	Pain flared up, and her left femoral pulse was present, but no distal pulses were palpable. Balloon angioplasty was performed throughout the SFA, and the culprit lesion was identified as the distal PES–IWS junction based on contrast pooling. To address this issue, a PES was implanted.
Day 10	Discharged
6 months follow-up	Ankle-brachial index was normal, and the patient was free from claudication.

## Case presentation

**Figure 1:**
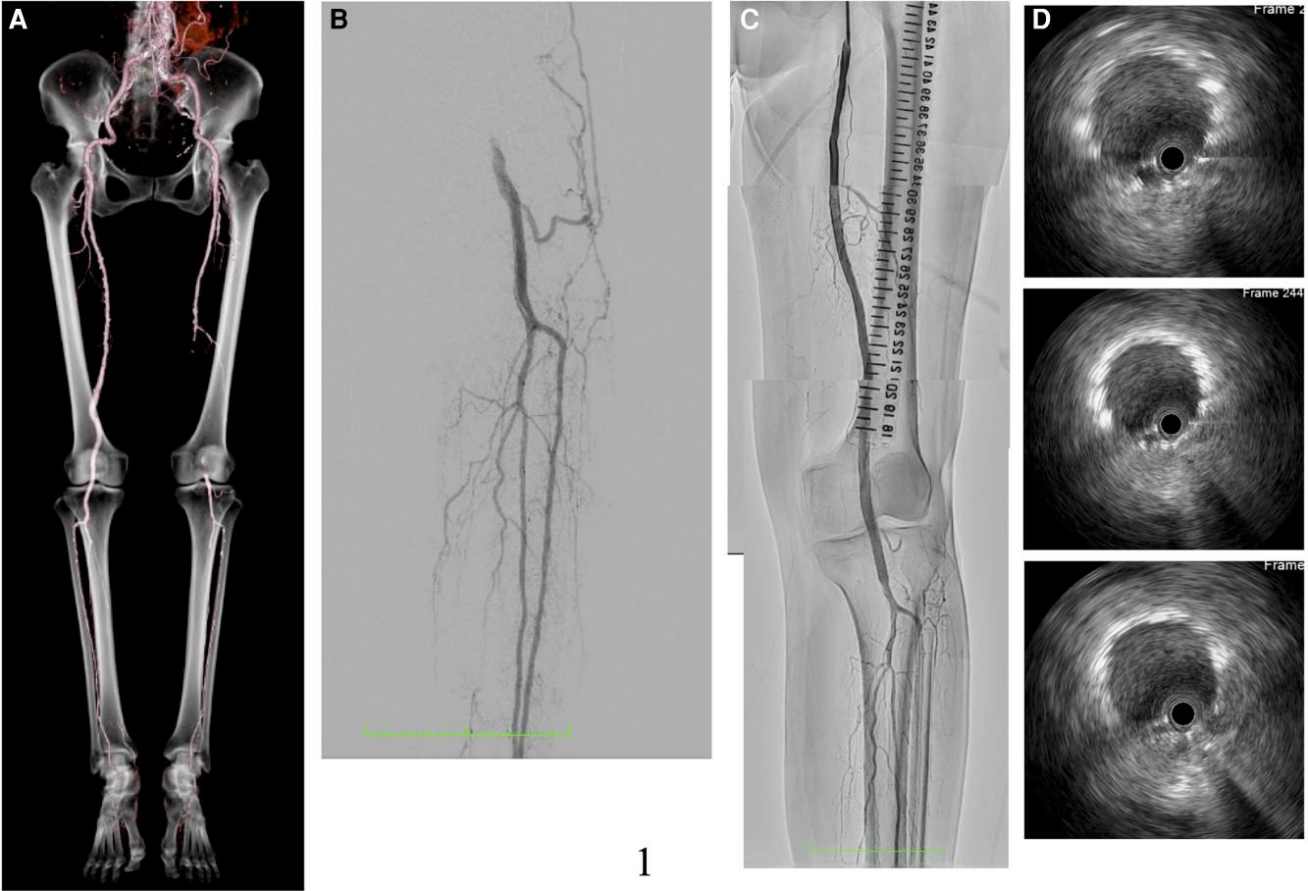
Computed tomography angiogram post-endovascular treatment (*A*). Control angiogram showed left popliteal artery occlusion from the left dorsalis pedis (*B*). The final angiogram revealed good blood flow from the superficial femoral artery down to the lesion below the knee (*C*). Intravascular ultrasound showed excellent expansion and apposition of the placed stents (*D*).

**Figure 2:**
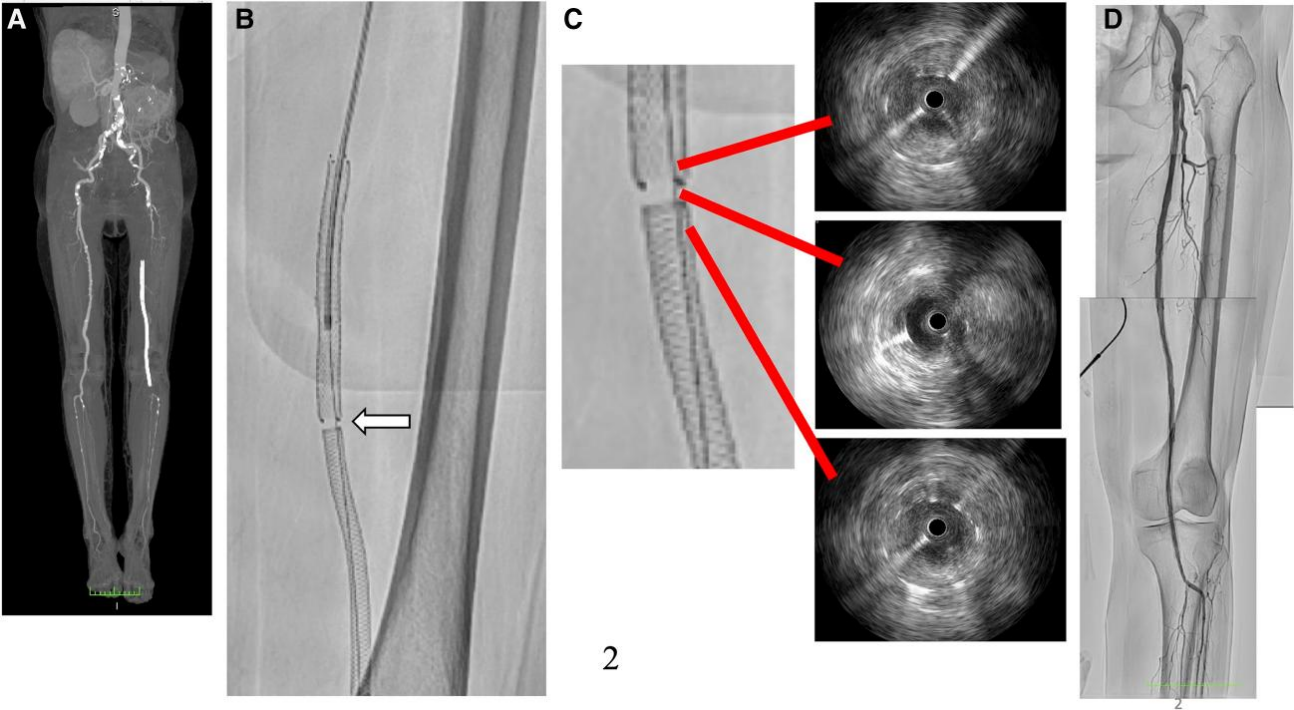
Computed tomography angiogram showed total occlusion from ostial superficial femoral artery to popliteal artery (*A*). The angiogram revealed a gap (white arrow) between the stents that were previously implanted (*B*). According to the intravascular ultrasound results, there was a significant amount of thrombus in the stents and evidence of neointimal hyperplasia in the gap (*C*). Successful revascularization of the superficial femoral artery to popliteal artery (*D*).

An 82-year-old woman presented with intermittent claudication in her left foot and an ankle-brachial index of 0.5. An enhanced computed tomography (CT) scan revealed a completely occluded 20 cm lesion in her superficial femoral artery (SFA) (*Figure 1A*). Despite medical therapy, her symptoms persisted, and she opted for endovascular treatment. A 5 Fr guiding sheath was inserted by puncturing the dorsalis pedis artery (*Figure 1B*), and 5000 units of heparin were administered. Attempts to cross the lesion with a 0.014-inch wire using a needle in the catheter failed due to severe fibrous sclerosis. A new access site was chosen, and a 6F guiding sheath was crossed by puncturing the contralateral common femoral artery (CFA). With an increased wire tip load of 15 to 45 g, the wire was finally able to pass through the chronic total occlusion (CTO) after thrombus aspirati*on and a 3.0 mm × 40 mm* balloon dilatation. The wire had almost passed into the intraplaque, and the healthy area in the popliteal artery was only the P3 area. Due to the lesion’s length, *a 6.0 mm × 150 *mm *interwoven stent (IWS)* and *a 7.0 mm × 80 mm paclitaxel-eluting stent (PES)* were overlapped from the popliteal artery to the SFA (*Figure 1C* and *D*). The patient was discharged the next day without complications. However, after 6 months, she reported left leg pain for 2 days, and an enhanced CT scan showed a complete absence of blood flow in the ostial SFA (*Figure 2A*), resulting in a diagnosis of acute limb ischaemia (ALI) (Class IIa). Endovascular therapy (EVT) was performed starting from the contralateral right CFA with a 6 Fr guiding sheath. Surprisingly, angiography revealed a gap between the two consecutive stents (*Figure 2B*, Supplementary material online, *Video S1*). Following aspiration, intravascular ultrasound (IVUS) imaging showed significant thrombus in the ostial SFA and neointimal hyperplasia in the gap (*Figure 2C*). To address this, we treated the gap with a 4.0 mm × 15 mm cutting balloon. Subsequently, blood flow was restored to normal (*Figure 2D*). She was started on aspirin 100 mg/day following a diagnosis of LEAD, and cilostazol 100 mg/day was added at the first EVT session. Aspirin was discontinued, and a direct oral anticoagulant (apixaban 10 mg/day) was started due to thrombotic lesions.

**Figure 3:**
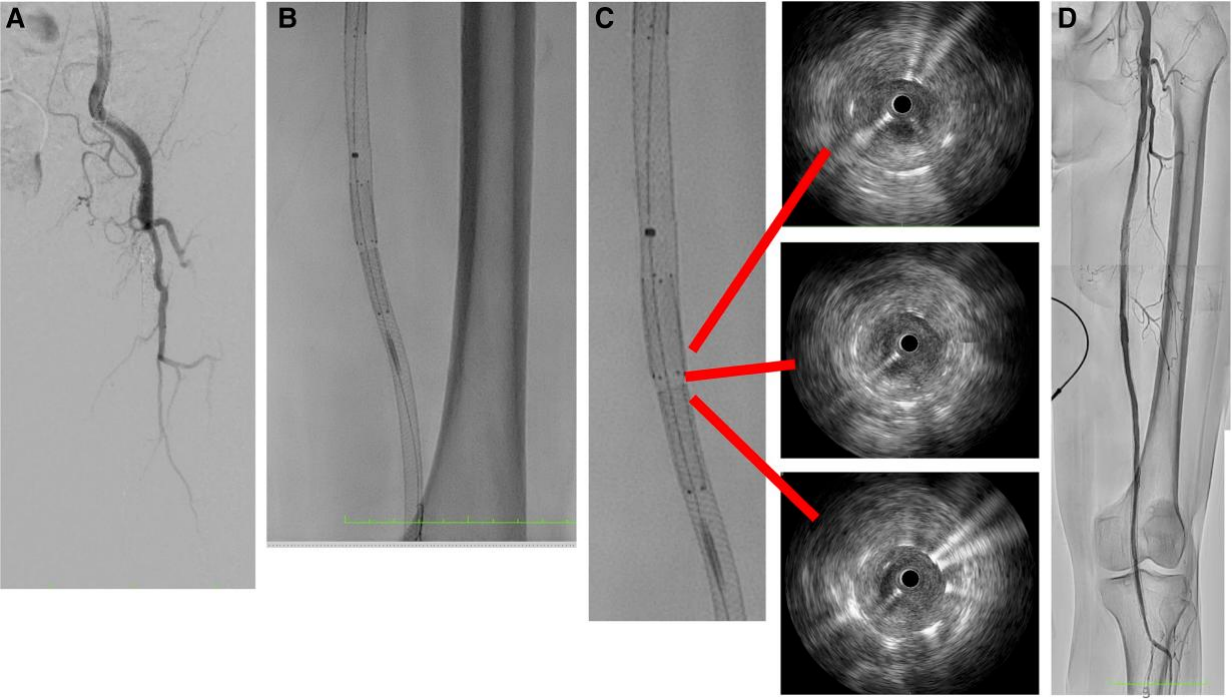
The initial angiogram revealed total occlusion of the superficial femoral artery ostium (*A*). In the gap, paclitaxel-eluting stent was implanted (*B*). Intravascular ultrasound showed good expansion and apposition of the overlap and gap lesion (*C*). The final result was a significant improvement in blood flow (*D*).

The next morning, her leg became cold with no palpable pulses in the common femoral to dorsalis artery. Her right leg was normal, and 12-channel electrocardiogram showed normal sinus rhythm. A duplex ultrasound revealed that there was no blood flow in her SFA ostium, requiring further treatment. A metal-tipped microcatheter successfully passed a 0.014-inch wire through the total occlusion of the SFA ostium (*Figure 3A*), and IVUS showed a significant thrombus. After thrombus aspiration, a 7.0 mm × 4.0 mm PES was implanted to push against the thrombus in the gap between the IWS and the PES (*Figure 3B* and *C*). The final angiogram showed complete revascularization (*Figure 3D*), and the patient was discharged 10 days later without recurrence symptoms and thrombotic occlusion.

Six months later, she kept coming to the hospital as an outpatient as if nothing had happened.

## Discussion

The treatment of long CTO lesions in the SFA is dominated by bypass surgery. However, bypass surgery is highly invasive and not recommended for high-risk patients. Currently, EVT is often the first choice due to its minimally invasive approach.^3,4^ For TransAtlantic Inter-Society Consensus II C and D lesions, multiple stents may need to be placed to cover the entire lesion since it is too long for a single stent to cover. Research demonstrates that full lesion covering stenting has better outcomes than spot stenting.^5^ The SFA region is subjected to unique mechanical influences that affect the surrounding muscles, including extension, torsion, and compression. In the popliteal artery, the primary mechanical influence is flexion.

Acute limb ischaemia is a sudden and severe symptomatic condition that occurs when there is reduced arterial blood flow. Causes can include the progression of occlusive lesions, cardiogenic emboli, arterial dissection, graft thrombosis, popliteal artery aneurysm, cystic adventitial degeneration of the popliteal artery, entrapment syndrome, trauma, hypercoagulable states, and medical complications.^1^ In our case, angiography at the time of her first ALI showed images of a gap between two stents, but there was a large amount of thrombus in the proximal part of the SFA. After aspirating the thrombus in the angiography images of the second ALI, there was an accumulation of contrast media in the gap between the two stents, the cause of decreased blood flow was determined to be the same site, and the PES was placed. There are two reasons to think that the gap is the cause: first, the blood flow in the knee was good after thrombus aspiration and runoff is not a problem; second, both stents were well expanded and crimped according to IVUS, and there was no fracture. The absence of recurrent ALI in this patient following stenting in the gap provides further evidence that the gap was the culprit lesion. Stenting in the popliteal artery is often avoided due to concerns about obstructing the bypass anastomosis, low primary patency, and stents fracture. However, recent real-world data suggest that an IWS implanted in the popliteal artery has a 1-year patency rate of 89.6% as well as a significantly higher radial resistive force compared to laser-cut stents.^6^ A comparative study of percutaneous transluminal angioplasty and stenting found similar 1-year patency rates for both treatments (65.8% vs. 58.7%).^7^ It is worth noting that stenting was used for bailout purposes in the latter study, which makes direct comparisons somewhat challenging. After reviewing the angiography of the first EVT, it appears that there is a 5 mm overlap between the stents. It was reported that if more than one stent is required, an overlap (between 0.5 and 1 cm) is considered acceptable.^8^ This guidance pertains to stacking stents of the same type of bare metal stent, and there are no established recommendations in the literature regarding the use of mixed stents. The IWS is implanted in a relatively elongated state at the time of deployment. It is possible that the shape memory alloy shortened during the chronic phase, causing the overlap portion to shorten. The presence of a stent gap in the coronary artery territory is reported to cause abnormal progression of neointimal hyperplasia.^9^ However, the incidence of a gap of the SFA has not been reported in the literature. Inadequate overlap of the IWS and the self-expanding stent may have led to IWS shortening and gap formation in the chronic phase, resulting in neointimal hyperplasia and recurrent thrombotic occlusions.

## Conclusion

A gap can form between overlapping stents, which can lead to ALI. Therefore, it is necessary to ensure that the overlap is sufficiently reduced when implanting the stents.

## Supplementary Material

ytad542_Supplementary_Data

## Data Availability

There are no new data associated with this article.

## Lead author biography

Yuki Matsumoto graduated from Iwate Medical University in 2012. He started training in Cardiology at Iwate Medical University from 2014. He now works in the Department of Cardiology at Ofunato Hospital.

## Supplementary material

**Consent:** Informed consent for patient information and images to be published was provided by the patients in accordance with COPE guidelines.

**Funding:** None declared.

## References

[1] Aboyans V , Ricco JB, Bartelink MLEL, Björck M, Brodmann M, Cohnert T, et al 2017 ESC guidelines on the diagnosis and treatment of peripheral arterial diseases, in collaboration with the European Society for Vascular Surgery (ESVS). Eur Heart J 2018;39:763–816. 29425606 10.1016/j.rec.2017.12.014

[2] Tomoi Y , Soga Y, Takahara M, Fujihara M, Iida O, Kawasaki D, et al Spot stenting versus full coverage stenting after endovascular therapy for femoropopliteal artery lesions. J Vasc Surg 2019;70:1166–1176. 30850285 10.1016/j.jvs.2018.12.044

[3] Aihara H , Soga Y, Mii S, Okazaki J, Yamaoka T, Kamoi D, et al Comparison of long-term outcome after endovascular therapy versus bypass surgery in claudication patients with Trans-Atlantic Inter-Society Consensus-II C and D femoropopliteal disease. Circ J 2014;78:457–464. 24292129 10.1253/circj.cj-13-1147

[4] Antoniou GA , Chalmers N, Georgiadis GS, Lazarides MK, Antoniou SA, Serracino-Inglott F, et al A meta-analysis of endovascular versus surgical reconstruction of femoropopliteal arterial disease. J Vasc Surg 2013;57:242–253. 23159476 10.1016/j.jvs.2012.07.038

[5] Tomoi Y , Takahara M, Kuramitsu S, Soga Y, Iida O, Fujihara M, et al Subintimal versus intraluminal approach for femoropopliteal chronic total occlusions treated with intravascular ultrasound guidance. J Am Heart Assoc 2021;10:e021903. 34612052 10.1161/JAHA.121.021903 PMC8751881

[6] San Norberto EM , Flota CM, Fidalgo-Domingos L, Taylor JH, Vaquero C. Real-world results of supera stent implantation for popliteal artery atherosclerotic lesions: 3-year outcome. Ann Vasc Surg 2020;62:397–405. 31449958 10.1016/j.avsg.2019.06.038

[7] Scheinert D , Scheinert S, Sax J, Piorkowski C, Bräunlich S, Ulrich M, et al Prevalence and clinical impact of stent fractures after femoropopliteal stenting. J Am Coll Cardiol 2005;45:312–315. 15653033 10.1016/j.jacc.2004.11.026

[8] Bildirici U , Aktas M, Dervis E, Celikyurt U. Mid-term outcomes of stent overlap in long total occluded lesions of superficial femoral artery. Med Sci Monit 2017;23:3130–3135. 28649980 10.12659/MSM.902413 PMC5498130

[9] Diletti R , Zijlstra F. Stent overlap in acute myocardial infarction. EuroIntervention 2017;13:e505–e507. 28781245 10.4244/EIJV13I5A79

